# Full-Scale Experimental Study of Groundwater Softening in a Circulating Pellet Fluidized Reactor

**DOI:** 10.3390/ijerph15081592

**Published:** 2018-07-27

**Authors:** Ruizhu Hu, Tinglin Huang, Aofan Zhi, Zhangcheng Tang

**Affiliations:** 1 Key Laboratory of Northwest Water Resource, Environment and Ecology, MOE, Xi’an University of Architecture and Technology, Xi’an 710055, China; www_lonely_com@163.com (R.H.); zhizhiing17@163.com (A.Z.); sdtangzhch@163.com (Z.T.); 2 Shaanxi Key Laboratory of Environmental Engineering, Xi’an University of Architecture and Technology, Xi’an 710055, China

**Keywords:** pellet reactor, circulating fluidization, groundwater softening, full-scale experiment

## Abstract

The softening effect of a new type of circulating pellet fluidized bed (CPFB) reactor on groundwater was studied through a full-scale experiment. The operation of the CPFB reactor in the second water plant in Chang’an District in Xi’an China was monitored for one year, and the results were compared with those for the Amsterdam reactor in The Netherlands. The removal efficiency of Ca^2+^ in the CPFB reactor reached 90%; the removal rate of total hardness was higher than 60%; effluent pH was 9.5–9.8; the turbidity of the effluent and the turbidity after boiling were lower than 1.0 NTU; the unit cost was less than €0.064 per m^3^; and the softened effluent was stable. The pellets in the CPFB reactor were circulated, providing higher crystallization efficiency. The diameter of the discharged pellets reached between 3–5 mm, and the fluidized area height of the CPFB reactor was 4 m. The performance parameters of the CFPB reactor were optimized.

## 1. Introduction

Pellet softening in a fluidized bed reactor was developed and introduced in the 1970s in The Netherlands [1]. Almost all of the drinking water in The Netherlands was conditioned in 2016 and approximately 50% was softened by pellet fluidized bed (PFB) reactors [2]. PFB reactors provide evident advantages over the lime or adsorption softening of water [3,4].

The Amsterdam reactor is currently the most widely used PFB reactor. The fluidized bed part is a part of the Amsterdam reactor, which is cylindrical with a height of approximately 5 m [5,6]. Seeds are placed at the bottom of the reactor and fluidized under upward flow. The calcium carbonate (CaCO_3_) crystallization on the seeds mainly occurs at the bottom of the reactor [6,7]. The pilot and full-scale study of the Amsterdam reactor on the removal of total hardness (TH) and Ca^2+^ and the development of the growth kinetics of pellets were carried out in the Weesperkarspel drinking water treatment and pilot plant of Waternet in Amsterdam, The Netherlands [2,8,9]. For example, Hofman et al. (2006) presented 20 years of experience with PFB reactor softening in The Netherlands. They reported that the pellets discharged from the reactor reached approximately 1.0 mm, and the removal rate of Ca^2+^ was approximately 50% [1]. Van Schagen et al. (2008) adopted mathematical models to show that the pellet size control in a PFB reactor had a significant influence on performance with respect to the water quality parameter. Maintaining the pellet size at the bottom of the reactor at 0.8 mm instead of 1.4 mm reduced the supersaturation of CaCO_3_ in the water after the reactor by 50%. However, this increased the consumption of the seeding material by 550% [10]. Schetters et al. (2015) studied the reuse of ground pellets as seeding material in the pellet-softening process through a pilot-scale experiment. The effluent TH was in the range of 0.2 mmol/L to 2.0 mmol/L, and the pellet discharge diameter was only between 0.6–1.2 mm [8]. Chen et al. (2016) applied the Amsterdam reactor in recirculating cooling water softening, and analyzed the influence factors, including the pH, height of the fluid bed, particle size, influent flow, and reflux ratio (ratio of the part of the effluent flow refluxed to influent flow and influent flow) on hardness removal. The effluent concentration of Ca^2+^ reached a removal efficiency of 86.6% [11]. Hu et al. (2017) studied the influence of factors including superficial velocity (*SV*), particle size (*L*_0_), and supersaturation (*S*) on the pellet growth rate of CaCO_3_. In addition, they developed two models of pellet growth rate and fixed bed height growth rate in a pilot-scale experiment on the Amsterdam reactor, and reported that the pellet discharge diameter reached 1 mm to 2 mm [12].

It can be seen from the above-mentioned studies that the diameter of discharged pellets can only reach 1 mm to 2 mm, and the size of the pellets was uneven from the bottom to the top of a reactor. However, the size and distribution of the pellets in a PFB reactor can directly determine the crystallization efficiency and the resistance ability against hydraulic impact. Disturbed layers were observed frequently. Bed height depended on flow, and garnets were typically flushed out of the reactor [13]. The removal efficiency of TH and Ca^2+^ can be further improved. This paper introduces a circulating pellet fluidized bed (CPFB) reactor that helps the circulation growth of pellets based on the Amsterdam reactor. The pellet size tends to be uniform in the crystal growth process from the bottom to the top of the reactor. The CPFB reactor can effectively prevent the problem of uneven pellet size, extend the discharge time, improve the crystallization efficiency, reduce the reactor height, increase the discharge size of the pellet, and ensure the high removal efficiency of TH and Ca^2+^.

## 2. Materials and Methods

### 2.1. Material

The water source of the full-scale experiment was the groundwater of the second water plant in Chang’an District, Xi’an, China. Table 1 presents the water quality data, showing that the water is mainly characterized by temporary hardness, which is extremely suitable for PFB reactor softening. The turbidity after boiling should be considered as the standard for softening to ensure drinking water quality.

Garnets with a size range of 0.2 mm to 0.4 mm, and a density of 3.93 g/cm^3^, were used as seeds in pellet reactors. NaOH was adopted as a softening agent, and its mass concentration was 30% [12]. Hydrochloric acid (HCl) was used to adjust pH, and its mass concentration was 30%.

### 2.2. Full-Scale Experimental System

**Figure 1:**
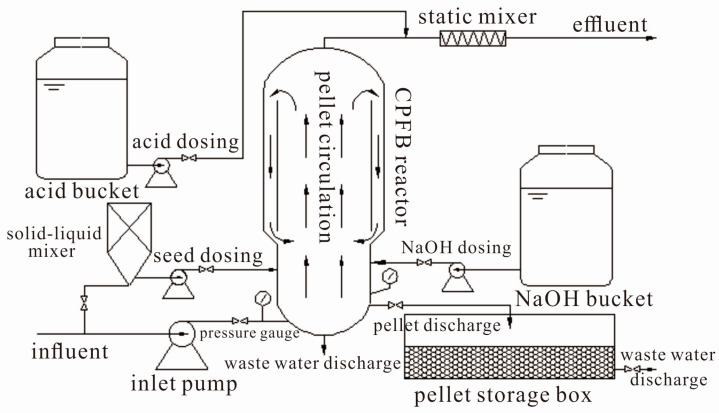
Schematic diagram of the full-scale pellet softening reactor.

Figure 1 shows the diagram of the full-scale pellet softening reactor. The softening system was mainly composed of influent and effluent systems, a NaOH dosing system, an acid-dosing system, a seed-dosing system, and pellet discharge and storage systems. The core of this system was the CPFB reactor, which produces 5000 m^3^ of soft water every day.

The CPFB reactor was different from the single layer cylinder structure of the Amsterdam reactor, which had a double cylinder structure and a different diameter for the upper and lower cylinders. The crystalline pellets can circulate to the bottom in the upper part of the inner cylinder. The structure design improved the growth efficiency of the pellets and stabilized the bed height.

The specific equipment parameters of the system are shown in Table 2. As shown in the table, the superficial velocity of the CPFB reactor was 60 m/h to 100 m/h, and the fluidized area height was 4 m, which was lower than that of the Amsterdam reactor (5–6 m) [6,14].

### 2.3. Experiment Process Description

High hardness groundwater was measured using an electromagnetic flowmeter, pumped into the CPFB reactor, and reacted with NaOH. A certain amount of garnet crystal seeds was pumped into the CPFB reactor after mixing with water through a pellet pump every day in normal operation. Mature pellets were discharged into the pellet storage box every day based on pressure change. Effluent pH was adjusted to 7–8 by HCl after the static mixer.

In the normal water supply process, superficial velocity was controlled by influent flow; then, NaOH dosage and acid dosage were adjusted manually based on influent flow. The hardness removal efficiency, the pressure change process in the CPFB reactor, and the growth kinetics of pellets were studied using water samples and pellet samples that were obtained at different heights of the CPFB reactor and at the outlet every few days. The system operation parameters are shown in Table 3.

### 2.4. Analysis Methods

The hardness and Ca^2+^ and Mg^2+^ concentrations of inlet water and outlet water was analyzed through ethylenediaminetetraacetic acid (EDTA) [15]. After drying, pictures of the pellets were taken using the microscope Nikon 50i (Nikon, Tokyo, Japan). The diameters of the pellets were determined by employing the American Society of Testing Materials (ASTM) sieving method [16]. The average pellet diameter was calculated by using Equation (1) [12]. The fluidized bed height after expansion was measured by a meter ruler. Pressure was monitored by an online pressure meter. pH was monitored using an online real-time pH meter and a handheld portable pH meter [10].

(1) $$\bar{d_{p}}=\frac{1}{\sum \frac{x_{i}}{d_{pi}}}$$

where $\bar{d_{p}}$ is the average diameter of the pellets in mm; $x_{i}$ is the mass fraction of the pellets trapped in the *i* sieve net layer; and $d_{pi}$ is the average diameter of the pellets trapped in the *i* sieve net layer in mm.

The information about the influent flow, inlet pressure, fluidized bed pressure, and effluent pH required in the experiment was collected in real time and transmitted to the central control room.

## 3. Results and Discussion

### 3.1. Experimental Study on NaOH and HCl Dosage Optimization

**Figure 2:**
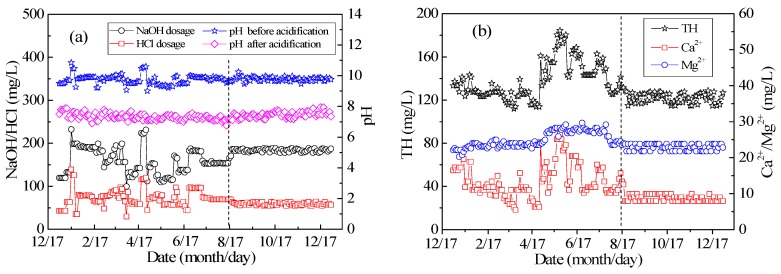
(**a**) Change in NaOH/HCl/pH and (**b**) TH and Ca^2+^/Mg^2+^ concentrations with time.

Figure 2a shows the variation in the NaOH and HCl dosages and effluent pH with time during operation in 2017. The data can be divided into the experimental data collection stage in months one to eight, and the steady system operation stage in months eight to 12. It can be seen from the figure that effluent pH increased with NaOH dosage, and the pH after adjustment decreased as the HCl dosage increased. In the normal system operation stage, the NaOH dosage was 180 mg/L; the HCl dosage was 50 mg/L, and the pH value can be adjusted to be stable between seven and eight.

Figure 2b depicts how the TH and Ca^2+^ and Mg^2+^ concentrations of the CPFB reactor effluent changed with time. The CPFB reactor evidently removed TH and Ca^2+^, with removal rates reaching 60% and 90% at 18–21 °C, respectively. According to the dosage and removal rate data, the removal of 1 mM Ca^2+^ needed 2.6 mM NaOH. As expected, the pellet softening process reduced the CaCO_3_ content in the water, and left the Mg^2+^ concentration unchanged [1], because the removal of Mg^2+^ must ensure that the pH exceeds 11 [17]. The pH of the CPFB reactor was controlled between 9.5–10 (Figure 2a).

**Figure 3:**
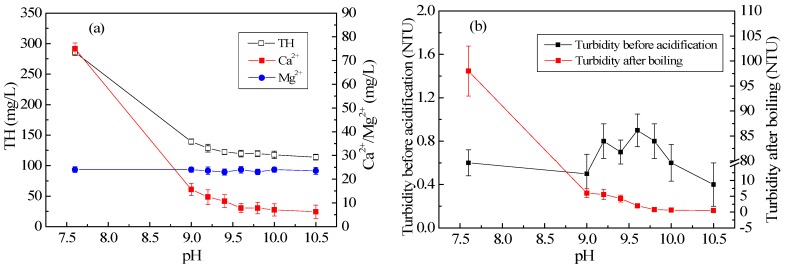
(**a**) Variation in TH and average concentrations of Ca^2+^ and Mg^2+^ with pH and (**b**) Variation in turbidity before acidification and turbidity after boiling with pH.

Figure 2 shows that the CPFB reactor ran smoothly, and the removal rates of TH and Ca^2+^ slightly fluctuated. Thus, the TH of the effluent, the average concentration of Ca^2+^ in the effluent, the turbidity of the effluent, and the turbidity after boiling under different NaOH dosage conditions can be calculated using the data shown in Figure 2. Figure 3 illustrates the direct relationship between the effluent pH and the ionic concentration and turbidity. Thus, the crystallization and hardness removal effects of the CPFB reactor can be predicted by monitoring the effluent pH, which was useful for automatic control and effluent quality prediction. Figure 2 and Figure 3 depict that the CPFB reactor exhibited a good and stable removal effect for hardness when the NaOH dosage was 180 mg/L (pH reaches 9.7) and HCl dosage was 50 mg/L (pH reaches 7–8). It also ensured that the effluent turbidity and the turbidity after boiling were less than 1.0 NTU.

Various reports presented that the softening effect of the PFB reactor is not exactly the same during operation because of the different drinking water quality standards in different countries and different softening purposes such as drinking and scale inhibition. At the same time, the removal rate of calcium and hardness is affected not only by the crystallization process, but also by the dosage of softeners, the types of softeners, and alkalinity. However, the literature has rarely reported on the CPFB reactor providing removal rates of 90% and 60% for Ca^2+^ and TH, respectively. For example, Hofman et al. (2006) presented the water quality parameters of raw and treated water with 20 years of operation experience in Waternet, Vitens and Brabant Water and found a Ca^2+^ removal rate of less than 50% [1,18]. Hammes et al. (2011) reported that the “Amsterdam-type” pellet softening reactor could reduce Ca^2+^ concentration from 1.65 mM to 0.8 mM; the removal efficiency was only 50% [14]. Therefore, the CPFB reactor has significant advantages in water softening, which can provide data references for engineering with a high Ca^2+^ or TH removal rate.

### 3.2. Experimental Study on Pellet Distribution and Hardness Removal Characteristics at Different Heights of CPFB Reactor

**Figure 4:**
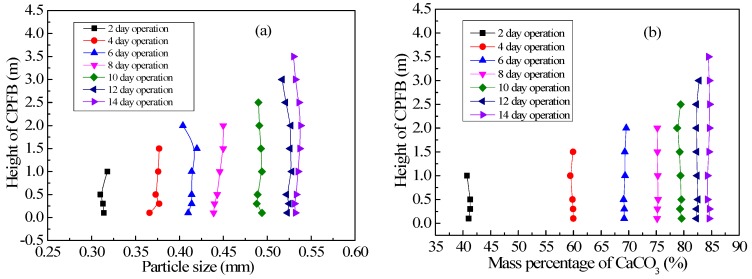
(**a**) Granularity distribution and (**b**) the mass percentage of CaCO_3_ at different heights of the CPFB reactor.

Figure 4a,b shows the relationship between the average pellet size distribution and the mass percentage of CaCO_3_ at different heights of the CPFB reactor with an increase in operation time. Pellet size became consistent at different heights of the CPFB reactor as the operation time increased, which is considerably different from the literature, where the pellet diameter in the Amsterdam PFB reactor gradually decreased from the bottom to the top. The pellet size at the bottom can reach 1–2 mm. However, the pellet size at the top was only 0.2–0.3 mm. These pellets were not yet crystallized [10,14]. The pellet size distribution state was affected by the water flow fluctuation. If influent flow increases abruptly, the higher velocity will take small pellets out of the PFB reactor.

**Figure 5:**
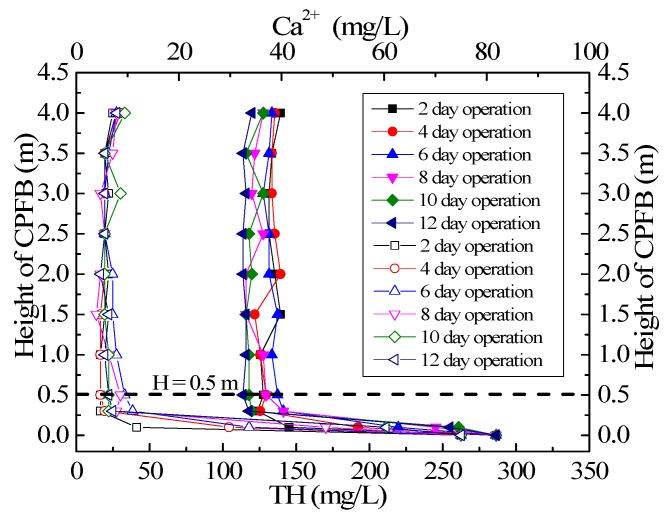
Removal of TH and Ca^2+^ at different running times of the CPFB reactor.

The pellet size distribution in the CPFB reactor tended to be uniform because of its double cylinder structure design, which can make the circulating pellets grow. The special pellet circulation structure of the CPFB reactor resulted in a different growth kinetics of the pellets compared to the Amsterdam reactor. In the CPFB and Amsterdam reactors, crystallization occurred mainly at the bottom of the reactor [7,19]. As shown in Figure 5, more than 90% of TH and Ca^2+^ were removed below 0.5 m in the CPFB reactor. The water and the chemicals mixed at the bottom of the reactor; thus, the pellets at the bottom of the reactor crystallized first. However, the pellet location in the Amsterdam reactor remained relatively stable under hydraulic screening; thus, the pellet size at the bottom rapidly increased. In the CPFB reactor, the small pellets at the top fell to the bottom under different density flows, then, they were flushed up by the flow. The CaCO_3_ crystallization was in progress when the pellets were flushed up. The pellet size at the bottom continuously increased, the voidage in the fluidized zone increased, and the pellets were constantly poured into the circulation zone for growth. Finally, the pellet size of the entire fluidized zone tended to be uniform, which was why the average pellet size and the mass percentage of CaCO_3_ in the CPFB reactor were the same at different heights after operation for two weeks, as shown in Figure 4.

Therefore, the unique structure of the CPFB reactor increased the utilization of the seed material, extended the discharge time of the mature pellets, and importantly, reduced the height of the CPFB reactor. Due to the increase in crystallization efficiency, more than 90% of the hardness was removed below 1.0 m in the CPFB reactor, while the Amsterdam reactor can only remove 75% below 1.5 m [14]. Therefore, the height of the fluidization zone of the CPFB reactor was only 4.0 m, and it was likely to decrease further. The height of the Amsterdam reactor was mostly 5–6 m.

### 3.3. Experimental Study on Pellet Growth

**Figure 6:**
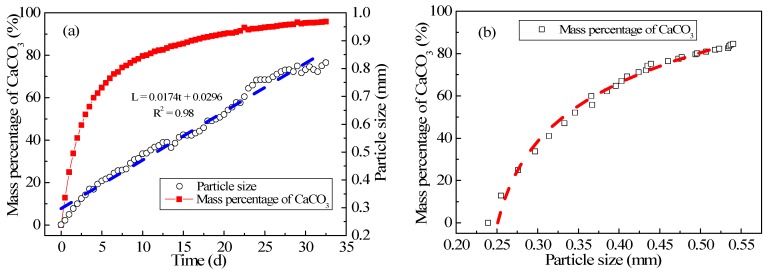
(**a**) Change in the average pellet size and the percentage of CaCO_3_ with running time at the bottom of CPFB reactor, and (**b**) Relation between the average pellet size and the percentage of CaCO_3_.

Figure 6a shows the relationship between the average pellet size and the mass percentage of CaCO_3_ when the CPFB reactor has just started to run. The average pellet size and the mass percentage of CaCO_3_ were positively related to running time. However, the growth rate of the average pellet size was the same, while the rate of increase in the mass percentage of CaCO_3_ presented a slower trend with running time. This is mainly because there is a relationship between the increase in the pellet size and the mass of the crystallized CaCO_3_. The mass of the crystallized CaCO_3_ was the same every day under the condition that the influent flow and hardness of the inlet and outlet water were stable. However, the increase in the mass percentage of CaCO_3_ of every pellet was related to the crystallization efficiency of CaCO_3_ for each pellet. Accordingly, the crystallization efficiency decreased as the pellet size increased [15]. However, note that a certain function relationship existed between the pellet size and the mass percentage of CaCO_3_, as shown in Figure 6b [10,20].

From Figure 6a, it can be seen the average pellet size was only approximately 0.8 mm when the reactor had run for 30 days. The crystallization efficiency of pellets was relatively high, and the mass percentage of CaCO_3_ increased. The first discharge time was considerably prolonged. As described in Section 2.2, pellets can grow cyclically in the CFPB reactor; therefore, the size of pellets was less at the bottom of the reactor. The pellet growth rate can be calculated by fitting the growth curve of the pellet size, and it was approximately 2.01 × 10^−10^ m/s, which was not less compared with that in the literature [12,16,21,22]. The functional relationship between the average pellet size and the mass percentage of CaCO_3_ can be obtained from Figure 6b. Thus, when one of the variables is known, it can be used to calculate another variable [5].

### 3.4. Experimental Study on the Relationship between Pressure and Bed Height Variation and Pellet Discharge

**Figure 7:**
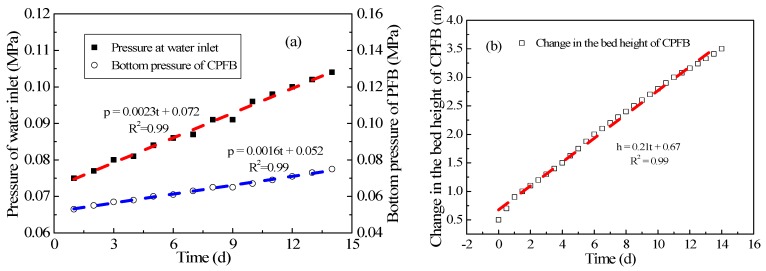
Change in (**a**) pressure and (**b**) bed height with operation time.

Figure 7a shows the change in inlet pressure with time. The increase in influent pressure (p) was directly proportional to time (t); the functional relation can be fitted as p = 0.0023 t + 0.0720 (R^2^ = 0.99). Similarly, the increase in the bottom pressure (p) was proportional to time (t), and the functional relation can be fitted as p = 0.0016 t + 0.052 (R^2^ = 0.99). These two formulas indicate that the influent pressure changed by 2.3 kPa daily, and the bottom pressure changed by 1.6 kPa. The influent pressure and bottom pressure increased with the operation time because of the continuous crystallization of CaCO_3_ on the crystal seeds every day. Based on the hardness of the influent and effluent, crystallization mass can be calculated to be approximately 430 kg/day, which can produce a pressure of approximately 2.1 kPa at the bottom of the CPFB reactor. The change in the pressure at the bottom of the reactor was the same as that obtained through fitted data.

The change in the bed height of the PFB reactor was examined in a few studies [9,16,19]. Figure 7b shows the change in bed height with time. The increase in bed height (h) was directly proportional to time (t), and the data can be fitted to h = 0.21 t + 0.67 (R^2^ = 0.99). The change in the bed height of the PFB reactor can be calculated to be approximately 0.21 m/day through the formula, which was coincident with that observed in the actual operation.

**Figure 8:**
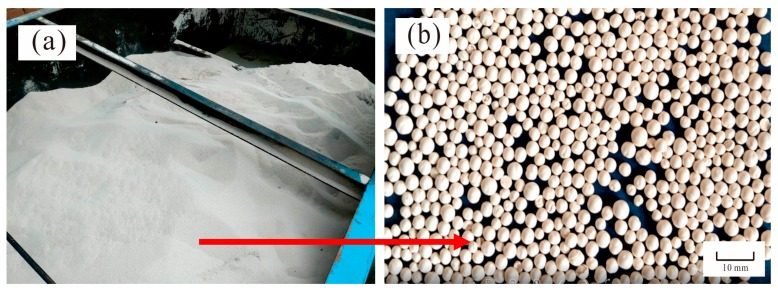
(**a**) Pellet storage box and (**b**) mature discharged pellets.

The actual pressure change and bed height change laws of the CPFB reactor can be obtained through Figure 7. The pellet discharge was closely related to the two change laws during the actual operation. In the early stage of CPFB reactor testing, the balance between the pellet discharge size and the bed height change must be found, and the pressure range of the CPFB reactor can also be determined at this time. The pellet discharge began when the pressure exceeded a certain value; when pressure is below a certain value, the pellets discharge stopped, and crystal seeds began to dose. The dosage of crystal seeds was calculated based on the daily discharge. The control mode of the pellet discharge and the dosage of crystal seeds were similar to those of the Amsterdam reactor [13]. However, owing to the circulatory crystallization of pellets in the CPFB reactor, the size of the discharged pellets was larger, as shown in Figure 8. Most of the pellet sizes in the CPFB reactor during normal operation were approximately 3–5 mm, which was considerably higher than the size of the discharged pellets for the Amsterdam reactor (1–2 mm) [7,14,23]. Importantly, the softening effect was ensured under high crystallization efficiency. The performance was better than that reported in the literature, where small white pellets appeared in the effluent when the pellet discharge diameters >1.1 mm [8]. However, as seen from Figure 8b, the pellets were not particularly uniform, mainly because the size of a few pellets increased until the flow could not suspend them in the process of pellet circulation, and these pellets will always crystallize at the bottom, leading to oversizing (larger than 5 mm). Therefore, it is necessary to discharge oversized pellets as far as possible during the pellet discharge process to prevent the effect of oversized pellets on the water quality.

### 3.5. Costs

Pellet softening on a large scale is relatively inexpensive. This process is more expensive when it is applied at a smaller scale, such as the majority of groundwater treatments in The Netherlands [1]. As described in the literature [1], the average cost for the large-scale treatment is €0.02 per m^3^ (101 million m^3^/year), but the average cost can be increased to at least €0.25 per m^3^ when the Amsterdam reactor is applied at a smaller scale. For the CPFB reactor softening system, the annual cost for the treatment of 5000 m^3^/day (installed capacity) mainly includes chemical cost, energy cost, and other costs. Other costs consist of the garnet cost and the labor cost, etc., which can be neglected. The specific cost analysis can be seen in Table 4.

As shown in the table, the cost of unit water treatment is € 0.058 per m^3^, while the chemical cost (NaOH + HCl) is 83% of the total cost, and the labor cost is only 4%. These values are significantly different from the cost composition of the Amsterdam reactor provided in the literature, in which the chemical and labor costs account for 32% and 25% of the total cost, respectively. This difference is mainly caused by the low labor cost in the water treatment plants of China. Even though the variations in cost are primarily caused by the variations in the market prices of NaOH and HCl, the water treatment cost of the CPFB reactor softening system is still lower than that of the Amsterdam reactor [1,8].

## 4. Conclusions

This study investigated the performance parameters of a CPFB reactor using a full-scale experiment system. The following conclusions were obtained:

1. In the CPFB reactor, the removal rate of Ca^2+^ and TH can reach 90% and 60%, respectively, and the effluent pH can be controlled between 9.5–9.8. The turbidity of the effluent and the turbidity after boiling are stable at less than 1.0 NTU. The unit water treatment cost is less than €0.064 per m^3^. The CPFB reactor has advantages in terms of the softening effect and cost.
2. The unique structure of the CPFB reactor improves the crystallization efficiency, increases the utilization of the seed material, and extends the discharge time of mature pellets. The size of the discharged pellets can reach 3–5 mm, and the height of the CPFB reactor is reduced from between 5–6 m to 4 m.

## Figures and Tables

**Table 1:** Groundwater quality.

No.	Water Quality	Value
1	pH	7.6–7.7
2	Temperature/°C	18–21
3	Turbidity of raw water/NTU	<1
4	Turbidity after boiling/NTU	90–100
5	Total alkalinity (CaCO_3_)/(mg/L)	262
6	Bicarbonate alkalinity (CaCO_3_)/(mg/L)	262
7	Total hardness (TH) (CaCO_3_)/(mg/L)	286
8	Ca^2+^ (mg/L)	75
9	Mg^2+^ (mg/L)	24

**Table 2:** Equipment parameters. CPFB: circulating pellet fluidized bed.

No.	Equipment Name	Parameter	Remarks
1	Pipeline pump	H = 0–25 m, Q = 200 m^3^/h, P = 15 kW	Frequency conversion pump
2	CPFB reactor	D = 1.6 m, H = 4.0 m	Stainless steel
3	Acid bucket	V = 12 m^3^	Polyethylene, design for 7 days
4	NaOH bucket	V = 12 m^3^	Polyethylene, design for 10 days
5	Pellet storage box	V = 15 m^3^	Carbon steel

**Table 3:** Operation parameters.

No.	Parameter Name	Value
1	Superficial velocity/m/h	60–100
2	NaOH dosage/mg/L	38–150
3	HCl dosage/mg/L	16–80
4	Pellet discharge/kgCaCO_3_/day	300–400
5	Garnet dosage/kg/time/day	25–50
6	pH before acidification	9.5–9.9
7	pH after acidification	7.0–8.0

**Table 4:** Cost analysis.

No.	Cost Composition	Euro	Percentage (%)
1	NaOH	69,228	65
2	HCl	19,230	18
3	Garnet	1154	1
4	Energy	13,461	12
5	Labor	3846	4
6	Total cost	106,919	100
7	Unit cost (€ per m^3^)	0.058	/
